# Genesis, Procedures, Attrition Rate and Major Reasons for Missing Measurement Session by the Study Participants in the Ellisras Longitudinal Study

**DOI:** 10.3390/children7060051

**Published:** 2020-05-27

**Authors:** Phuti Makgae, Betty Sebati, Hlengani Siweya, Kotsedi Monyeki

**Affiliations:** 1 Physiology and Environmental Health Department, University of Limpopo, Sovenga 0727, South Africa; phutimakgae@gmail.com (P.M.); bettysebati800@gmail.com (B.S.); 2 Executive Dean Faculty of Science and Agriculture, University of Limpopo, Sovenga 0727, South Africa; hlengani.siweya@ul.ac.za

**Keywords:** genesis, attrition rate, South African population

## Abstract

The noncommunicable diseases’ (NCDs) profile is changing rapidly from one country to another. A well-formulated cohort study in Africa could answer major questions relating to the changing profile of NCDs risk in Africa. The aim of the present study was to outline the genesis, procedures, attrition rate and major reasons for study participants to miss measurement sessions in the Ellisras Longitudinal Study (ELS). Method: The ELS followed multiple longitudinal designs comprising repeated measurements in more than one cohort with overlapping ages. Age cohort and time of measurement effects could be identified. A cluster random sampling method was used to sample 2255 participants (1201 males and 1054 females), aged 2 to 10.9 years at baseline (November 1996). Information on lifestyle (tobacco and smoking, alcohol intake, physical activity and socioeconomic status) and biological risk factors for NCD and educational achievements were collected over time. The participants were followed 17 times over the past 25 years with measurements (blood pressure and anthropometry) collected twice during the first consecutive 8 years to account for growth dynamics and other health-related variables. The attrition rate for ELS sample for boys (14%–27.3%) was significantly (*p* < 0.05) higher than girls (7.9%–18.6%) from May 1999 to November 2003. There was a significant (*p* < 0.05) increase (25.3%–70.3%) in attrition rate from November 2009 to December 2015. The ELS participant migration to urban areas provided a unique opportunity to investigate the effect of urban life on these rural young adults given the previous data collected on the same subjects at a younger age (3–10 years at baseline in 1996). Conclusion: A well-formulated ELS study in Africa could answer major questions relating to the changing magnitude of NCDs risk factor profiles in Africa.

## 1. Introduction

Globally, Africa is expected to experience the highest increase in noncommunicable diseases (NCD) related mortality, with about 46% of all expected mortality attributed to NCDs by 2030 [1]. It is increasingly recognised that the occurrence of adult NCDs are influenced by factors operating from childhood, which are sustained throughout the individual’s life course [2]. The increased risk may start in infancy or even before birth and will continue to be influenced by health-related behaviour during adulthood [1,3], yet evidence to capture these changes in NCD profile is still very limited in Africa. The South African National Development Plan (SANDP) vision for 2030 [4] highlights key recommendations for reducing the prevalence of NCD by 28% in 2030, which are all mirrored in the World Heart Federation, though they focus on reducing the NCDs prevalence by 25% in 2025 [5].

The desire of the South African government to improve the health of rural people requires that adequate baseline data be provided to combat the emerging NCDs as they are becoming a major health burden in communities today [4]. However, Twisk and Kemper [6] pointed out that growth and development studies, namely, cross-sectional, longitudinal and time-lag designs are influenced by several factors. The study design measurements are taken on a subject at a particular point in time and are influenced by the age of the subject, birth cohort to which the subject belongs, time of measurements, learning or test effects and the dropout effects [7,8,9,10]. These factors introduce the differences between repeated measurements and play a significant role in the analysis, interpretation and conclusion of the outcome measures of these studies. The study aimed to outline the genesis, procedures, attrition rate and major reasons for study participants to miss measurement sessions in the Ellisras Longitudinal Study (ELS).

## 2. Materials and Methods

### 2.1. Geographical Area

Ellisras is a rural area in the northwestern region of the Limpopo province, South Africa. It consists of 42 settlements with a population of about 50,000 people [11]. The majority of the workforce is in the mining sector and a few in education and civil services. Unemployment and poverty remain a challenge in this region [12].

### 2.2. Study Design and Sampling

Ellisras Longitudinal Study followed a multiple Longitudinal Study design suggesting that repeated measurements were carried out in more than one cohort with overlapping ages [13].

Using STATA, the sample size required for the study was calculated based on a power of 90% and a two-tailed significant level of 5%, the prevalence of undernutrition of 25% amongst rural South African populations [14,15] if the true prevalence or proportion of undernutrition is 22%. An estimated sample size of 2115 was required.

The study was undertaken at 22 schools (10 preschool and 12 primary schools) randomly selected from a total of 68 schools within the Ellisras area. Birth records were obtained from the principals of each school. Only those birth records that had been verified against health clinic records were used to determine the age of each potential participant. Each of the 22 chosen schools were assigned an age category (i.e., 3, 4, … 9, 10), and only children whose verified age was within the category for a particular school were assessed. Sample sizes by age ranged from 14 to 199 for boys and 17 to 174 for girls. Sample sizes per age group fluctuated within and between genders because within each school, there were different numbers of children of the designated age.

A total of 2225 (1201 boys, mean age 6.81 years, and 1024 girls, mean age 6.81 years, (age 3–10 years) SD 1.57) were followed from baseline throughout the periodic surveys (1996–2015). A total of 1701 subjects (873 boys, mean age 13.9 years, SD 1.92, and 828 girls, mean age 14.0 years, SD 1.87) were measured in November 2003, while in the last survey of November 2015, only 728 subjects (356 males and 372 females aged 24 years) who are part of the ELS study were found.

Table 1 presents research question data collected in the Ellisras Longitudinal Study Sample as from 1996 to Dec 2013. This was collected twice yearly from baseline to November 2003. Furthermore, information on lifestyle (tobacco and smoking, alcohol intake, physical activity and socioeconomic status) and biological risk factors for NCD and educational achievements were collected over time (Table 1). The questionnaire covered important concepts relating to lifestyle risk factors and socioeconomic status, which were previously validated with other populations and were also validated in the Ellisras population and subsequently used for data collection (also used in the current study [16,17,18]).

Blood samples were collected for the first time at the 17th point of data collection in November 2015. The participants were followed 17 times in the past 25 years with measurements (anthropometry and blood pressure) collected twice during the first consecutive 8 years to account for growth dynamics and other health-related variables (Table 1) in this Ellisras rural population.

### 2.3. Data Collection

All the schools that nested the ELS sample based on the baseline data were visited in February/March each year from 1997 to date. In each school/community, a contact person was established through the help of the school principal. The contact person/ELS coordinator was responsible for providing information regarding the whereabouts of the ELS participants in that particular school/village such that when the data collection resumed in May and November each year, ELS data forms for each ELS subject was located to the school of migration.

### 2.4. Ethical Clearance

Ethics approval was obtained from the University of Limpopo’s research ethics committee in the circle of 5 years from when the project started (TREC/356/2017:PG and MREC/P/204/2013IR). Parents and guardians were provided with and signed informed consent. Children at a younger age were also requested to sign an assent form after study information was provided. Later, the ELS participants took the responsibility of signing the consent form after information regarding the study was provided to them and all the question they had adequately answered by the principal investigator.

### 2.5. Quality Control

All selected ELS coordinators attended refresher training each year before the completion of the register at the beginning of the year. A 1-week intensive training was arranged in November 2003 in preparation of the completion of the questionnaire based on reasons for missing measurements session by the ELS subjects in November 2003. The questionnaire was completed after the measurement session of November 2003. The intertester (between fieldworkers) and intratester (principal investigator and field workers) technical error of measurements ranged from 97% to 100% in agreement with the coding of the respondent based on the pilot survey in the Ellisras area before the actual survey.

### 2.6. Statistical Analysis

Descriptive statistics of the mean age of the total number of ELS subjects in each period of measurement was recorded over time. Frequency and percentage frequency of ELS subjects missing the measurement period were recorded each year. Frequency and percentage frequency of the educational level, reasons for school dropout, employment level and family size of the ELS participants were reported. Frequency and percentage frequency of the educational level, reasons for school dropout and employment level of ELS participants was reported in November 2003. Student *t* test was used to determine the significant difference between gender, whereas the chi-square test was used to test the significant association between frequency in each gender. Fisher exact test was used to test the significant association between genders for frequency cell less than 10. Analysis of variance was used to test the significant difference between subjects missing measurement session over time. Bonferroni post hoc test was used to identify the period of measurement session showing a significant increase or decrease. All analyses were performed using the SPSS Version 14.0 (SPSS Inc., Chicago, IL, USA). *p*-value was set at *p* < 0.05.

## 3. Results

Table 2 presents the mean age and attrition rate from May 1997 to November 2015 overtime for ELS participants. The mean age ranges from 6.9 to 23.7 years for boys and 6.8 to 23.9 for girls throughout the measurement period (November 1996 to November 2015).

Table 3 demonstrates the analysis of variance and Bonferroni post hoc test for the significant difference between attrition rates in the measurement period of May 1997 to November 2015 overtime for the ELS participants. The attrition rate for ELS sample for boys (14%–27.3%) was significantly (*p* < 0.05) high than girls (7.9%–18.6%) during the period May 1999 to November 2003. There was a gradual insignificant increase (6.7%–27.3%) in the attrition rate during the period November 1997 to May 2003. There was a significant (*p* < 0.05) increase (25.3%–70.3%) in the attrition rate during the period November 2009 to December 2015.

Table 4 presents the frequency and percentage frequency of educational level, employment level and reasons for missing measurement session as reported on November 2003 amongst ELS participants. There was a significant (*p* < 0.05) high percentage (7.8%) of ELS girls who attended tertiary education as compared to boys (3.2%) in November 2003. The majority of the ELS sample (84.6%) were in secondary education level by November 2003. Furthermore, a majority of the ELS sample (55%) cited a lack of finance or school uniform or school fees and poverty as their main reasons for dropping out of school in November 2003. The level of unemployment (77%) for the ELS participants was high.

Table 5 presents the frequency and percentage frequency of family size of ELS participants as reported by November 2013. There was no significant (*p* < 0.05) difference in the family size for boys and girls across the family size categories. ELS participants reported a larger family sample of more than six family members (35.9% for girls and 32.7% for boys).

## 4. Discussion

The study aimed to outline the genesis, procedures, attrition rate and major reasons for sample missing during measurement session in the ELS. A total of 2225 children were sampled at baseline in the ELS using a cluster random sampling technique. This sample size was high at baseline and was similar from birth to 20 studies [16,19,20]. However, the Amsterdam Growth and Health Longitudinal Study sample size was low at baseline (1977) compared to our ELS [7,8].

The attrition rate of the ELS participants increased with increasing age, though insignificant, thus, reaching a significant rate at older samples measured from November 2009 to November 2015. Similar findings were reported in the Amsterdam Growth and Health Study [2,20]. This was because subjects completed their school years and were going for tertiary education, while others migrated to urban areas looking for work. Amongst ladies’ pregnancy played a significant role due to government funding strategies (child support grant from the government) for all young children under the age of 16 years [21]. The ELS participants migration to urban areas provided a unique opportunity to investigate the effect of urban life on these rural young adults given the previous data collected on the same subjects at a younger age (3–10 years at baseline in 1996). It is worth mentioning that a majority of the subjects returned home (Ellisras) in December each year, and they availed themselves for the ELS measurement session. Furthermore, poverty was cited as a major reason for dropping out of school.

Challenges of cohort studies in Africa were previously documented, of which, ELS is not an exception [19,22]. These include: long-term financial commitment; long-term commitment of staff and subjects, changing leadership in an institution where the study is housed may have negative consequences for continuity and marriage of subjects and migration to urban areas. All these affect the follow-up measurement of the subjects. Gordis [23] and Kemper [2] reported that in a cohort study, subjects who missed the measurement sessions should be included in the analysis to check if outcome of interest affected them in the previous analysis including all other subjects who are currently enrolled in the study. That will assist in reducing the subject bias which may affect the interpretation of the results and the conclusion drawn thereof. This was carried out with success in the previous analysis of ELS [24,25].

ELS has some limitations and strengths. Like all cohort studies, ELS participants are not self-selective, whereas in the Internet-based cohort epidemiological studies, participants tend to be self-selective and are feasible [26]. The ELS study, like all other population-based cohort studies, is expensive with changing methodology over time [27]. However, ELS provides unique data for the rural South African population for tracking of one biological risk factor to another. Knowledge of the level of tracking of a characteristic further helps to answer the question of whether or not lifestyle intervention should be provided for the whole population or to a subsample. If stability is high, the value of a character is a good predictor for that characteristic later in life [28].

One serious drawback of a Longitudinal Study is the number of subjects lost in time from the original sample. In the first 8 years (1996–2003), the main reason for withdrawal from the study was leaving school, pregnancy, illness, impregnating women, looking for work and death. In that period, the total percentage of dropout in the Longitudinal Study ranged from 2.4% to 23.3% [25]. However, permanent dropout groups due to death for anthropometric measurements, blood pressure and nutritional variables did not show any significant difference compared to those that are currently in the study [29]. ELS provides a unique baseline for tracking the changes of NCDs in rural South African population over time. Given the introduction of biochemical blood sample collection late in the ELS sample and the fact that most of the ELS samples are now from adults, it will be important to compare the current samples with the dropout for any outcome of interest to avoid biases in the interpretation and conclusion of the results [2,23].

## 5. Conclusions

Ellisras Longitudinal Study followed a multiple Longitudinal Study design rooted in cluster random sampling techniques. The attrition rate was low during the first 8 years of the study and increased as the sample grew older. A well-formulated ELS study in Africa could answer major questions relating to the changing magnitude of the NCDs risk factor profile in Africa.

## Figures and Tables

**Table 1:** Research question data collected in the Ellisras Longitudinal Study Sample as from 1996 to Dec 2013

Survey	1996	1997	1998	1999	2000	2001	2002	2003	2005	Dec 2013
Anthropometrical measurement	*	**	**	**	**	**	*	**		
Blood pressure				**	**	**	*	*		
Diet				*		*				
Socioeconomic status				*						
Glucose tolerance						*				
Learning environment						*	*			
Educational achievement (Maths and English)						*	*	*		
Aptitudes tests (IQ test)								*		
Tanner Scale							*	*		
Questionnaire on menarche								*		
Physical activity							*			
Fitness					*	*	*	*		
Smoking									*	*
Alcohol and drugs										*

* one survey, ** two surveys.

**Table 2:** Ellisras Longitudinal Study sample mean age and attrition rate during the period of measurements May 1997 to Nov 2015 over time.

	Males				Females				Number of ELS Subjects Over Time					
Period of Measurements	Age in Years	(SD)	Min	Max	Age in Years	SD	Min	Max	Males		Females		Total	
									n	%	n	%	n	%
Nov 1996	6.9	(1.57)	2.6	10.9	6.8	(1.57)	2.0	10.7	1201		1054		2255	
May 1997	7.3	(1.61)	2.8	11.3	7.3	(1.57)	2.4	11.2	1106		969		2075	
Missing									95	7.9	55	5.2	150	6.7
Nov 1997	8.1	(1.93)	2.9	11.8	8.2	(1.85)	2.9	11.9	1146		1054		1890	
Missing									55	4.5	0	0	55	2.4
May 1998	8.6	(1.95)	3.4	12.3	8.7	(1.84)	3.4	12.3	999		891		1890	
Missing									202	16.8	133	12.6	335	14.9
Nov 1998	9.1	(1.95)	3.9	12.8	9.1	(1.84)	3.9	12.8	1007		894		1901	
Missing									194	16.2	130	12.3	324	14.4
May 1999	9.7	(1.91)	4.4	13.3	9.7	(1.81)	4.4	13.3	1033		941		1974	
Missing									168	14.0*	83	7.9*	251	11.1
Nov 1999	10.2	(1.88)	4.9	13.8	10.2	(1.79)	4.9	13.8	998		920		1918	
Missing									217	18.1*	117	11.1*	334	14.8
May 2000	10.6	(1.91)	5.4	14.4	10.7	(1.81)	5.4	14.3	1006		918		1924	
Missing									195	16.2*	106	10.1*	301	13.3
Nov 2000	11.0	(1.90)	5.9	14.8	11.0	(1.82)	5.9	14.8	936		877		1813	
Missing									265	22.1*	147	14.0*	412	18.3
May 2001	11.5	(1.90)	6.4	15.3	11.6	(1.83)	6.4	15.0	962		904		1866	
Missing s									239	19.9*	120	11.4*	359	15.9
Nov 2001	12.0	(1.91)	6.9	15.9	12.1	(1.82)	6.9	15.6	914		855		1769	
Missing									287	23.9*	169	16.0*	456	20.2
May 2002	12.4	(1.91)	7.3	16.3	12.5	(1.87)	7.4	16.3	890		823		1713	
Missing									311	25.9*	201	19.1*	512	22.7
May 2003	13.4	(1.91)	8.3	17.3	13.5	(1.85)	8.4	17.1	942		878		1820	
Missing									259	21.6*	146	13.9*	405	18.0
Nov 2003	13.9	(1.92)	8.9	17.8	14.0	(1.86)	8.9	17.8	911		828		1739	
Missing									328	27.3*	196	18.6*	524	23.2
Nov 2009	15.3	(2.03)	10.2	18.3	15.3	(1.99)	10.3	19.2	854		800		1654	
Missing									347	28.9	224	21.3	571	25.3
Nov/Dec 2013	23.7	(2.05)	18.2	27.6	23.9	(2.06)	18.3	27.6	520		541		1061	
Missing									681	56.7	483	45.8	1164	51.6
Nov/Dec 2015	23.7	(2.01)	19.2	29.1	23.9	(2.01)	18.7	29.9	356		372		728	
Missing									845	70.3	652	61.9	1497	66.4

SD = Standard deviation, * = *p* < 0.05

**Table 3:** Analysis of variance for the significant difference between attrition rate during the period May1997 to Nov 2015 over time for the Ellisras Longitudinal study sample.

	Boys
Measurement Session	May97 Nove97 May98 Nove98 May99 Nove99 May00 Nove00 May01 Nove01 May02 May03 Nov03 Nov09 Nov13 Nov15 _____ns______ ______________________ _____________________________________ns ___________ ______________ns__________ _______ns_____ ___________ns_________________
	Girls
Measurement Session	May97 Nove97 May98 Nove98 May99 Nove99 May00 Nove00 May01 Nove01 May02 May03 Nov03 Nov09 Nov13 Nov15 ____________________________________ns__________________________________________________ ________ns___________ _____ns__

**Table 4:** Frequency and percentage frequency of the educational level, reasons for school dropout and employment level of Ellisras Longitudinal study participants reported as of Dec 2013.

	Total N = 1061		Males N = 520		Females N = 541	
	%(n)		%(n)		%(n)	
Educational level						
Primary school	9.6	(102)	10.4	(54)	8.9	(48)
Secondary school	84.6	(898)	86.0	(447)	83.4	(451)
Tertiary education level	5.7	(61)	3.7 *	(19)	7.8 *	(42)
Reasons for leaving school						
No money/uniform/school fees/no food at home	54.8	(581)	60.4 *	(314)	49.4 *	(267)
No reason given	1.0	(11)	0.6	(3)	1.5	(8)
Employed	2.3	(24)	2.7	(14)	1.8	(10)
Educational system in South Africa changing/no water in the community	0.7	(7)	0.2	(1)	1.1	(6)
Failed one grade many times/suffering	10.9	(116)	10.2	(53)	11.6	(63)
Did not get along with the teachers/not supported by my parents	0.7	(7)	1.2	(6)	0.2	(1)
Sick (epilepsy and eye)/pregnant or impregnate a lady/not having anybody to look after my child	8.4	(89)	5.4 *	(28)	11.3 *	(61)
Friends/peer pressure/marriage	1.1	(12)	1.2	(6)	1.1	(6)
Completed	7.0	(74)	5.4	(28)	8.5	(46)
Still at school	13.2	(140)	12.9	(67)	13.5	(73)
Employments level						
Labour	18.4	(195)	22.3 *	(116)	14.6 *	(79)
Professional	8.2	(87)	8.7	(45)	7.8	(42)
Unemployed	73.4	(779)	69	(359)	77.6	(420)

* *p* < 0.05.

**Table 5:** Frequency and percentage frequency of family size of Ellisras Longitudinal Study participants as of Dec 2013.

	Total, n = 1061		Male		Female	
	%(n)		%(n)		%(n)	
Less than three	28.5	(302)	31.7	(165)	25.3	(137)
More than three but less than six family members	37.2	(395)	35.6	(185)	38.8	(210)
More than six family members	34.3	(364)	32.7	(170)	35.9	(194)
